# Effects of eye patches on corneal ulcer healing and weight gain in stocker steers on pasture: a randomized controlled trial

**DOI:** 10.1093/tas/txab162

**Published:** 2021-09-23

**Authors:** Gabriele U Maier, Josh S Davy, Larry C Forero, Heejung Bang, Kristin Clothier, John A Angelos

**Affiliations:** 1 Department of Population Health and Reproduction, University of California, Davis, CA 95616, USA; 2 Agriculture and Natural Resources Tehama, Glenn and Colusa counties, University of California, Red Bluff, CA 96080, USA; 3 Agriculture and Natural Resources Shasta and Trinity counties, University of California, Redding, CA 96002, USA; 4 Department of Public Health Sciences, Division of Biostatistics, University of California, Davis, CA 95616, USA; 5 California Animal Health and Food Safety Lab, Davis, CA 95616, USA; 6 Department of Medicine and Epidemiology, University of California, Davis, CA 95616, USA

**Keywords:** cattle, corneal ulcer, eye patch, infectious bovine keratoconjunctivitis, pinkeye, weight gain

## Abstract

Infectious bovine keratoconjunctivitis (IBK) is a painful ocular disease in cattle that is characterized by the presence of a corneal ulcer and production losses. A common industry practice is to cover an affected eye with a piece of cloth to reduce exposure to face flies and ultraviolet light with the goal of alleviating pain, accelerating healing, and reducing spread. To study the efficacy of eye patches in the treatment of IBK, a group of 216 clinically normal Angus crossbred steers were followed between April and August 2019 and evaluated weekly for the development of IBK. Eyes of cattle that developed IBK were enrolled with a blocked randomization scheme based on ulcer severity score to receive either an eye patch (treatment group) or no eye patch (control group). All treatment and control group animals received parenteral antimicrobial and nonsteroidal anti-inflammatory treatments and were housed in a pasture separated from the rest of the cohort for a maximum of 28 d or until clinical cure. Corneal ulcer areas were measured, and body weights were recorded twice weekly for steers in the treatment and control groups. Weights of all steers in the cohort were recorded three times during the trial period. The primary outcome, rate of corneal ulcer healing, was higher (*P* = 0.001) for lesions in eyes receiving an eye patch as determined by a linear mixed model that controlled for ulcer severity score at enrollment and previous IBK in the opposite eye. Median corneal ulcer healing time was 10 (IQR [Interquartile range] 7–17) d for patched eyes vs. 14 (IQR 7–21) d for unpatched eyes. In a Cox proportional hazards model adjusted for severity score at diagnosis, the hazard ratio for ulcer healing was 1.62 (95% CI: 1.02–2.56, *P* = 0.042) for eyes that received a patch compared to eyes that did not. Among all 216 steers in the cohort, those that were diagnosed with IBK had a numerically higher average daily gain (ADG) (0.45 [±SE 0.01] kg) vs. those that were not (0.42 [±SE 0.12] kg; *P* = 0.06). In enrolled steers that received a patch, the secondary outcome ADG was 0.47 (±SE 0.02) kg compared to 0.43 (±SE 0.02) kg in controls (*P* = 0.22). Weight gain may have been confounded by pasture during the treatment period. Results of this trial support the use of this low-cost intervention; further investigation into possible reasons for observed differences in weight gain may be warranted.

## INTRODUCTION

Infectious bovine keratoconjunctivitis (IBK), commonly known as pinkeye, is an infectious ocular disease of cattle that negatively impacts productivity and welfare and is characterized by conjunctivitis and corneal ulcers (Baptista, 1979). Data on incidence and prevalence are sparse; however, incidence in cow-calf herds has been estimated to reach up to 45% in calves during the summer (Dennis and Kneipp, 2021). Affected animals display epiphora and blepharospasm, signs that are commonly associated with pain or discomfort (Williams, 2010). Although some cases of IBK heal spontaneously (Alexander, 2010), the disease carries the potential for complete and permanent vision loss due to rupture of the cornea (Williams, 2010). Apart from the impact on welfare that comes with the disease, IBK also represents an economic burden due to reduced body weights, estimated between 15 and 30 lbs, in calves at weaning (Thrift and Overfield, 1974) as well as the cost of antibiotic treatments (McConnel et al., 2007) and additional labor for treatment of cases. There are several etiologic agents that have been associated with IBK, among them *Moraxella bovis, Moraxella bovoculi* (Angelos, 2015), and *Mycoplasma bovoculi*; the latter may act in synergy with *Moraxella* spp. to cause the disease (Schnee et al., 2015). Due to the fastidious nature of these agents and the large number of secondary bacteria which flourish during IBK infections, recovery of the primary pathogens from affected eyes can be challenging and false-negative results can occur. The face fly (*Musca autumnalis*) is considered a fomite responsible for the spread of bacterial species between cattle (Alexander, 2010) and may also contribute to corneal damage while feeding on eye secretions, predisposing to IBK (Shugart, 1978). Ultraviolet light, dust, and plant awns are additional risk factors that may damage the integrity of the corneal surface and thus predispose cattle to IBK.

Treatment options for IBK include the use of antimicrobial drugs administered intramuscularly, subcutaneously, subconjunctivally, or topically. Many different antibiotics appear to be effective for the treatment of IBK (Angelos, 2015). However, there has been increasing concern that the continued use of antimicrobials in food producing animals may lead to resistance of bacterial pathogens to antimicrobials in animals (AVMA, 2020) and possibly in humans. The National Action Plan for Combating Antibiotic Resistance states as one of its goals the identification and implementation of measures to develop non-traditional therapeutics and innovative strategies to minimize outbreaks caused by resistant bacteria in human and animal populations (The White House, 2015).

Fly control measures or other ancillary interventions such as applying an eye patch to an affected eye are commonly used nonantimicrobial adjuncts to prevention or treatment; however, quantitative data on the efficacy of these measures are sparse to nonexistent. Eye patches could provide multiple benefits for affected animals including relief from exposure to visible and ultraviolet light, relaxing the iris and reducing ciliary-body spasm, thereby diminishing pain, and shielding the eye from access to face flies, which could reduce the spread of the infection amongst herd mates. These benefits may also lead to increased weight gain because the animal is more likely to continue grazing.

The main objective of this study was to evaluate the efficacy of eye patches for reducing corneal ulcer size over time as well as time to heal in cattle with naturally occurring IBK. A secondary objective was to compare weight gain between groups of IBK-affected cattle that received a standard treatment regimen with or without application of an eye patch. The hypotheses were that corneal ulcer sizes will diminish more rapidly, healing times will be faster, and weight gains will be greater in steers that are treated for IBK with an eye patch vs. those that do not receive an eye patch. Data on the efficacy of preventative and supportive measures to combat IBK in cattle will be helpful in formulating best practices beyond treatment with antimicrobial or anti-inflammatory drugs.

## MATERIALS AND METHODS

### Study Population

The protocol for this trial was approved by the Institutional Animal Care and Use Committee at UC Davis (protocol # 20995). Angus crossbred steers (*n* = 216) with a mean body weight of 225 ± SD 25 kg obtained after an overnight fast at the start of the study were followed between April 23 and August 20, 2019, for the development of IBK. Steers were pastured at the University of California Sierra Foothill Research and Extension Center near Browns Valley, California. Historically, cattle at this research station have had an incidence of IBK of up to 50% during any given year. Steers arrived at this research facility approximately 1 wk prior to the start of the study and were vaccinated on arrival with a modified live injectable vaccine for infectious bovine rhinotracheitis, bovine viral diarrhea type 1 and 2 (Express 3, Boehringer Ingelheim, Duluth, GA), an intranasal vaccine for infectious bovine rhinotracheitis, parainfluenza-3 and bovine respiratory syncytial virus (Inforce 3, Zoetis, Parsippany, NJ), an injectable 8-way Clostridial vaccine (Ultrabac 8, Zoetis, Parsippany, NJ), an anaplasmosis vaccine (Louisiana State University, Baton Rouge, LA), were treated for intestinal parasites (Dectomax, Zoetis, Parsippany, NJ), and were implanted with a trenbolone acetate and estradiol ear implant (Revalor-G, Merck Animal Health). Throughout the trial, steers received a custom mix free choice loose mineral supplement (A. L. Gilbert Co., Oakdale, CA). None of the steers had received a pinkeye vaccine prior to study start nor did they wear insecticide impregnated ear tags during the trial period. All steers were weighed again on August 13, 2019, and at the end of the grazing season on October 17, 2019, after being kept off feed and water overnight.

### Experimental Design

Once per week, all steers were gathered and observed for clinical signs of IBK including epiphora, blepharospasm, or corneal opacity by having them walk single file through an alley with multiple observers on either side. Steers that were suspected of having IBK were separated and more closely examined in a hydraulic squeeze chute. Eyes of suspect animals exhibiting corneal opacities suggestive of IBK were stained with fluorescein sodium strips (Jorgensen Laboratories, Loveland, CO) to determine if a corneal ulcer was present. A diagnosis of IBK was made based on the presence of a corneal ulcer. Corneal ulcers that appeared to have been caused by mechanical trauma as evidenced by stellate or oblong-shaped areas of fluorescein sodium uptake were not enrolled.

**Figure 1.**
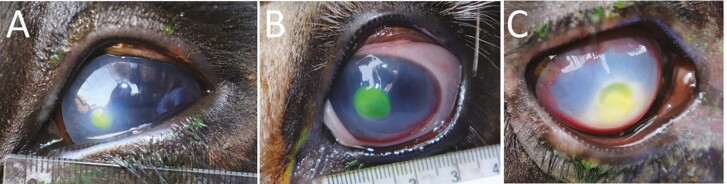
Examples of severity scores 2 (A), 3 (B), and 4 (C) assigned at enrollment in a trial evaluating time to healing for corneal ulcers caused by infectious bovine keratoconjunctivitis between those steers randomly assigned eyepatches at diagnosis and a control group not assigned eye patches. Score 2: lesion (ulcer and corneal edema) covers up to one-third of corneal surface area; score 3: lesion covers up to two-thirds of corneal surface area; score 4: lesion covers the entire corneal surface or eyeball is ruptured.

Ulcers were assigned a severity score based on the following criteria: 0 = no ulcer seen; 1 = widest diameter of ulcer ≤ 2 mm (pinpoint ulcer); 2 = lesions (ulcer and corneal edema) affecting up to one-third of cornea; 3 = lesions (ulcer and corneal edema) affecting up to two-thirds of cornea; and 4 = lesions involving the entire cornea or ruptured eyeball (Figure 1). Steers with a score of 2 or greater were randomly assigned (using a random number generator function in Excel [Microsoft, Redmont, WA]) to either receive an eye patch or not within strata of severity scores 2, 3, and 4. Before treatments were administered, affected eyes were photographed. A ruler was held rostral to the eye while the image was taken to calibrate measurements performed with image analysis software (ImageJ, National Institutes of Health, Bethesda, MD). At enrollment, steers in both treatment and control groups received a subcutaneous injection of 20 mg/kg oxytetracycline (LA-200, Zoetis, Parsippany-Troy Hills, NJ) as well as an IV injection of 10 mg/kg flunixin meglumine (Banamine, Merck Animal Health, Kenilworth, NJ). A commercial cattle cloth eye patch (Shuteye, American Animal Health, Inc., Wisner, NE) was glued to the skin surrounding the orbit with glue adhesive supplied by the manufacturer leaving the lower rim of the patch unattached for drainage. Duct tape was applied at the patch edges to improve durability of attachment after initial patches that were only attached with glue were often observed to fall off shortly after application. If a steer required antimicrobial treatment for a different reason, such as pneumonia or injury, while their IBK lesion was being followed, only data until the timepoint of the additional treatment were included in the data analysis. Enrolled steers were kept in a separate pasture close to the cattle chute and were examined, weighed, and photographed twice weekly (Tuesdays and Fridays). If no improvement was seen on a subsequent visit, a subcutaneous dose of florfenicol (40 mg/kg, Nuflor, Merck Animal Health, Kenilworth, NJ) was administered. Enrolled animals were followed until ulcers were healed or for a maximum of 28 d following initial enrollment, whichever came sooner. Twenty-eight days were considered a timeframe when the majority of lesions should have healed based on vaccine studies that evaluated time to healing in unvaccinated controls (George et al., 2005; Angelos et al., 2012). Ulcers were considered healed when no fluorescein stain uptake could be detected. When IBK was detected in both eyes simultaneously, a coin flip determined which side was enrolled. A steer with a healed lesion in one eye was eligible to be re-enrolled with the opposite eye if the first lesion had completely healed at the time of a second ulceration event.

To determine if bacteria commonly associated with IBK were present in this herd, culture swabs were taken from several IBK cases and normal eyes throughout the study, and transported in Amies charcoal transport media to the California Animal Health and Food Safety Laboratory (Davis, CA) for culture. Swabs were inoculated onto 0.5% sheep blood 3% agar (3% SBA), incubated for 48 h at 35 ± 2°C in 5–10% CO_2_ and 0.5% SBA for 48 h at 35 ± 2°C in a microaerophilic atmosphere (6–12% O_2_ and 5–8% CO_2_.). Culture plates were examined every 18–24 h for *Moraxella* spp. suspect colonies, which were confirmed by biochemical testing and matrix-assisted, laser desorption-ionization time of flight (MALDI-TOF) mass spectrometry.

Corneal ulcers in the digital photographs of eyes were traced with a stylus on a computer tablet and the area of the ulcer was calculated with image analysis software (ImageJ, National Institutes of Health, Bethesda, MD; Angelos et al., 2010; Gard et al., 2016).Ulcers were traced three times and the mean of the three tracings was used in the analysis. Prior to ulcer tracing, images were calibrated with the use of the ruler included in each photo to account for differences in magnification between images.

The investigator diagnosing IBK and assigning a severity score at enrollment was unaware of which treatment would be assigned (patch or no-patch). Once a steer was enrolled, blinding of researchers was impossible given the visibility of the patch covering the eye. Likewise, in the digital images of ulcerated eyes, it was possible to see evidence of an eyepatch so that blinding of the individual performing ulcer tracings could not be guaranteed during the process of measuring corneal ulcer areas.

### Statistical Analysis

Statistical analyses were performed with SAS software version 9.4 (SAS Institute, Cary, NC). Baseline weight between treatment groups was compared with an unpaired t-test after visually assessing the distribution for normality on plots. For the primary outcome ulcer size over time as the response variable, linear mixed models were built with affected individual eye as the random effect, time since enrollment, treatment group, the interaction of time and treatment group (a parameter of primary interest that estimates different time-trends), as well as severity score at enrollment (Fitzmaurice et al., 2011). The outcome variable ulcer size underwent natural log transformation to ensure normal distribution of residuals on residual plots. At the last timepoint, when the ulcer was declared healed, 0.001 was added to ulcer sizes of 0 cm^2^ prior to transformation to be able to include those data points in the analysis. A sensitivity analysis was performed that excluded a potential outlier of a large ulcer due to a perforated cornea that was identified by visual inspection of a plot of all ulcer size trajectories. Time to healing between groups was assessed with a Kaplan–Meier plot and a log-rank test as well as a Cox proportional hazards analysis model. Time to healing was censored at 28 d, when an animal died, or when an animal was treated with antimicrobials for unrelated reasons.

For the secondary outcome weight gain, average daily gain (ADG) over the trial period of 177 d on pasture was compared between steers diagnosed with IBK during the trial vs. those that remained free of IBK and between those that received an eye patch for IBK treatment vs. the control group with ANCOVA (Vickers and Altman, 2001). Steers treated with antimicrobials for unrelated causes were excluded from the analysis for weight gain in a sensitivity analysis to evaluate confounding of weight gain by disease. The weight gain comparison between steers receiving a patch and those not receiving a patch excluded those that were enrolled with both eyes in discordant groups (once with a patch, once without a patch), but included those that were enrolled twice with a patch or twice without a patch. Furthermore, body weight change over individual treatment periods of a maximum of 28 d was compared between treatment groups in linear mixed models with animal as the random effect. Model fit for different covariance structures of mixed models was assessed using the Akaike Information Criterion (AIC). Parameters were estimated using the restricted maximum likelihood method and the Satterthwaite method was used to estimate degrees of freedom.

## RESULTS

The total number of enrolled IBK lesions included in the analysis was 96 with 20 of those lesions occurring in the opposite eye of previously identified lesions. Of the 20 cases occurring in opposite eyes, 9 were assigned a patch and 11 were assigned no patch. The distribution of severity scores among the 96 ulcers that were analyzed is described in Table 1. One enrolled lesion was eliminated after the second timepoint when the animal had developed pneumonia and was treated with tulathromycin and flunixin meglumine. One lesion was followed for 10 d at which point the animal required treatment with antibiotics for an unrelated injury and subsequent observations were eliminated from analysis. One steer died after the second time point from unrelated causes. One steer developed pinkeye in the opposite eye after he had been followed for 14 d and was enrolled with the opposite eye on that day at which point the first lesion, which was only 0.01 cm^2^, was no longer followed. Finally, the last enrolled steer was no longer followed after 14 d at study end. The minimum time to healing in enrolled cases was 3 d. Healing time was not measured beyond 28 d when 15 ulcers had not yet healed. Seven steers required treatment with florfenicol because of corneal ulcers that showed no improvement or appeared at risk of eyeball rupture due to severe IBK. Of the 7 receiving florfenicol, 3 had been assigned a patch and 4 had not been assigned a patch. Of the 13 corneal swabs of eyes without any signs of IBK that were cultured, 5 resulted in no growth, 7 grew a mixed flora, and 1 grew *Moraxella bovoculi.* Of the 20 corneal swabs of eyes with signs of IBK that were cultured, 2 resulted in no growth, 7 grew a mixed flora, 8 grew *Moraxella bovoculi*, and 3 grew *Moraxella bovis*.

**Table 1.** Number of cases of infectious bovine keratoconjunctivitis enrolled by severity score at baseline in a clinical trial testing the time to healing between those cases that received an eyepatch vs. those that did not in a group of 216 Angus cross steers between April and August 2019

Severity score	Patch	No patch	Total
2 = ulcer and edema affecting up to 1/3 of cornea	22	23	45
3 = ulcer and edema affecting up to 2/3 of cornea	17	18	35
4 = ulcer involving entire cornea or ruptured globe	10	6	16
Total	49	47	96

### Corneal Ulcer Size

**Figure 2.**
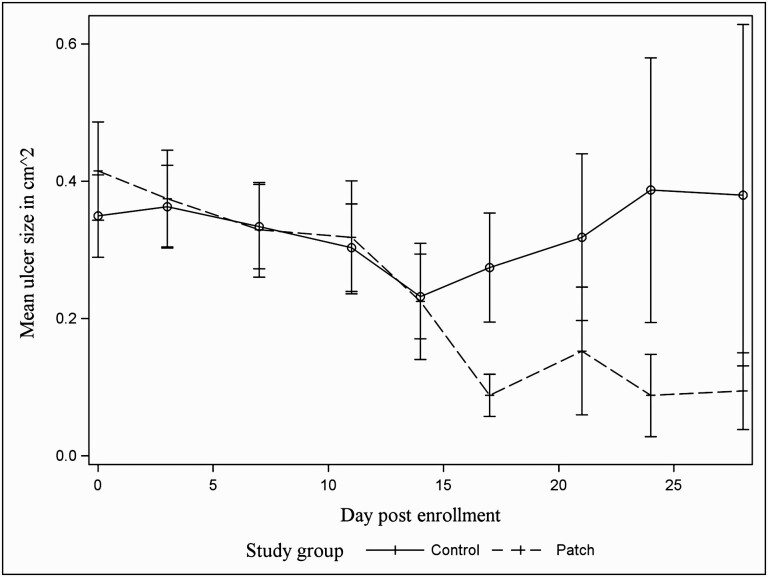
Mean ulcer size in cm^2^ (±SE) for 96 cases of infectious bovine keratoconjunctivitis in a group of 216 Angus crossbred steers followed until healing for a maximum of 28 d after diagnosis between April and August 2019. “Patch” group received an eyepatch covering the eye at diagnosis.

The mixed model with the natural log transformed outcome variable for ulcer size, random intercept for ulcer size, a time by treatment group interaction term, and the covariates for severity score at enrollment, and whether or not the opposite eye had already had pinkeye or not, showed that ulcer size decreased more rapidly for those steers that were assigned an eyepatch (*P* = 0.001; Figure 2 and Table 2). The covariance structure for the G matrix that minimized AIC was unstructured. The control group contained one steer with a ruptured cornea which resulted in a lesion with a large surface area during the entire follow-up period and could be regarded an outlier based on visual inspection of a spaghetti plot of all lesions. Data from this animal were therefore excluded in a sensitivity analysis, although the measurements taken were accurate. Model outcomes were similar, with a statistically significant interaction between treatment and days since enrollment (*P* = 0.009).

**Table 2.** Estimates for a linear mixed regression model on the effect of an eye patch assigned at diagnosis of infectious bovine keratoconjunctivitis (IBK) on ulcer size over time for 96 cases of IBK diagnosed in a group of 216 Angus cross steers between April and August 2019

Variable	N	Coefficient	Standard Error	*P*-value
Treatment				
Control	47	Ref.		
Eye patch	49	−0.289	0.366	0.44^*a*^
Days since enrolment		−0.141	0.013	<0.001
Treatment x Days since enrolment				
Control		Ref.		
Eye patch		−0.0635	0.020	0.001
Severity score at enrolment^*b*^				
2	45	Ref		
3	35	1.59	0.37	<0.001
4	16	2.26	0.48	<0.001
Previous IBK in opposite eye				
No	76	Ref.		
Yes	20	0.93	0.38	0.01
Intercept		−2.72	0.32	<0.001

^*a*^ *P* for treatment indicates no difference at baseline, i.e., day 0, supporting successful randomization.

^*b*^Score 2: lesion (ulcer and corneal edema) covers up to one-third of corneal surface area; score 3: lesion covers up to two-thirds of corneal surface area; score 4: lesion covers the entire corneal surface or ruptured globe.

### Healing Time

Healing time was censored at 28 d when 15 ulcers had not yet healed completely. The median follow-up time for all lesions in the study was 14 (Interquartile range [IQR] 7–22.5) d. Median follow-up times by score and whether a patch was assigned for 76 lesions that healed during the follow up period are described in Table 3. Table 3 does not include the follow-up time for those 15 lesions that had not yet healed after 28 d and for 5 lesions that were not followed before ulcers were completely healed for various reasons as described above, but before 28 d of follow-up.

**Table 3.** Median time to healing for ulcers in a study comparing ulcer healing between lesions assigned an eyepatch or no eyepatch at the time of diagnosis in 76 lesions in crossbred Angus steers

Group		Median time to healing in days (IQR)^*a*^
Severity score 2^*b*^	Patch assigned, *N* = 21	7 (7, 10)
	No patch assigned, *N* = 19	11 (7, 17)
Severity score 3^*b*^	Patch assigned, *N* = 13	14 (7, 18)
	No patch assigned, *N* = 11	14 (10, 21)
Severity score 4^*b*^	Patch assigned, *N* = 7	18 (14,24)
	No patch assigned, *N* = 5	21 (17,21)
All lesions	Patch assigned, *N* = 41	10 (7. 17)
	No patch assigned, *N* = 35	14 (7, 21)

Follow-up time was right-censored at 28 d.

^*a*^Interquartile range.

^*b*^Score 2: lesion (ulcer and corneal edema) covers up to one-third of corneal surface area; score 3: lesion covers up to two-thirds of corneal surface area; score 4: lesion covers the entire corneal surface or ruptured globe.

**Figure 3.**
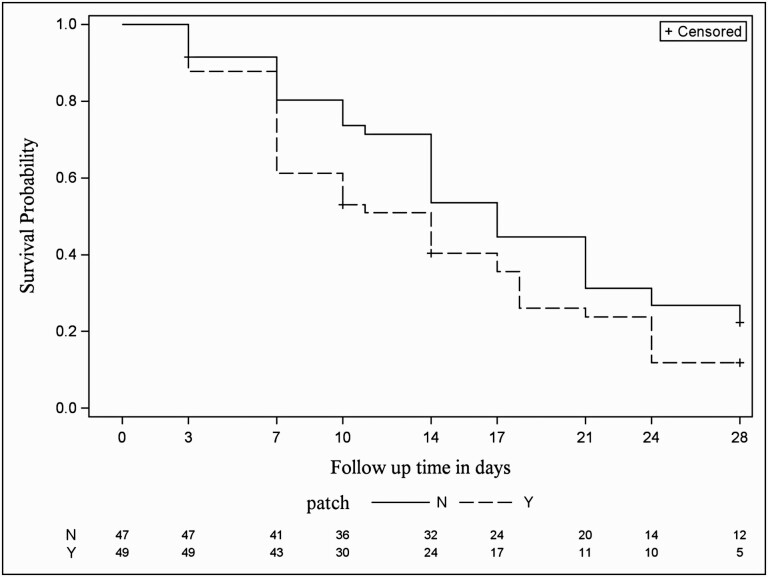
Kaplan–Meier curves showing time to healing for 96 infectious bovine keratoconjunctivitis ulcer lesions diagnosed in a group of 216 Angus crossbred steers followed between April and September 2019 by control vs. intervention. “Patch” group received an eyepatch covering the eye at diagnosis. Log-rank test *P* = 0.08.

In a Cox Proportional Hazards model of all enrolled lesions, adjusted for severity score at diagnosis, the hazard ratio for ulcer healing was 1.62 (95% CI 1.02–2.56, *P* = 0.042) for those steers assigned a patch compared to those that were not assigned a patch. The dichotomous variable for whether a previous lesion in the opposite eye had occurred was not statistically significant (*P* = 0.17) The Cox model is described in Table 4. The Kaplan–Meier curve showing time to healing for lesions with patches vs. no patches is shown in Figure 3. The log-rank test for the Kaplan–Meier curves yielded *P* = 0.08.

**Table 4.** Cox proportional hazards model for 96 steers in a trial evaluating time to healing for corneal ulcers caused by infectious bovine keratoconjunctivitis between those steers randomly assigned an eyepatch at diagnosis and a control group not assigned eye patches

Variable	*N*	Coefficient	Standard Error	Hazard Ratio	95% CI	*P*-value
Treatment						
Control	47			Ref.		
Eye patch	49	0.48	0.24	1.62	1.02–2.56	0.042
Severity score^*a*^						
2	45			Ref.		
3	35	−0.82	0.26	0.44	0.26–0.73	0.002
4	16	−0.96	0.34	0.38	0.20–0.74	0.005

^*a*^Score 2: lesion (ulcer and corneal edema) covers up to one-third of corneal surface area; score 3: lesion covers up to two-thirds of corneal surface area; score 4: lesion covers the entire corneal surface or ruptured globe.

### Weight Gain

The mean body weight (SD) of all steers (*N* = 216) at the beginning of the trial was 225 (± 24) kg. The mean weight after 112 d on pasture (*N* = 213) was 259 (±27) kg and the mean final weight after 177 d on pasture (*N* = 212) was 302 (±29) kg. The estimated ADG (SE) over the 177 d on pasture for all steers was 0.44 (±0.008) kg. For all steers that were treated for pinkeye at least once (*N* = 76), the ADG was 0.45 (±0.01) kg while it was 0.42 (±0.01) kg for those that had not been treated for pinkeye (*N* = 136; *P* = 0.06, adjusted for initial weight).

In enrolled steers that had received an eye patch, the ADG (SE) over the 177 d on pasture was 0.47 (± 0.02) kg while it was 0.43 (± 0.02) kg for those who did not receive an eye patch excluding six steers that were enrolled twice, once with and once without a patch. However, this difference was not statistically significant (*P* = 0.22).

Enrolled steers that received an eye patch had a similar mean weight (SE) on day of enrollment (242.5 [±4.3] kg) as compared to steers in the control group (243.2 [±3.9] kg; *P* = 0.90). There was no significant interaction between time and treatment groups for the weight change during the treatment period of up to 28 d while the IBK lesions were healing (*P* = 0.43).

A sensitivity analysis for weight gain outcomes excluding steers that had been treated with antimicrobials for unrelated causes led to qualitatively similar results.

## DISCUSSION

To the best of our knowledge, this is the first assessment of the efficacy of eye patches on healing time of ulcers and weight gain in cattle suffering from IBK in the published literature. Ulcer sizes shrunk faster in the group that received eye patches vs. the control group when controlling for severity score at enrollment and whether pinkeye had been diagnosed in the opposite eye. The Kaplan–Meier curves evaluating time to healing only tended to be significantly different; however, the analysis did not control for severity score at enrollment, which may be an important factor in ulcer healing. Time to healing was shorter in the eye patch group vs. the control group after controlling for severity score at enrollment in a Cox proportional hazard analysis.

We left eye patches in place until ulcers were healed or for a maximum of 28 d, which may not correspond to practices on commercial ranches. From our study, it is not known whether eye patches would also lead to more rapid ulcer healing if they were only used during the initial 10 d following identification of an ulcer, but further research would be needed to ascertain whether this is the case. It is possible that the first days are more critical when corneal edema and inflammation may result in temporary blindness in the affected eye. Such temporary blindness may lead to exacerbation of injury to the cornea caused by an animal with impaired vision hitting its blind eye on objects in its environment. The eye patch may provide a mechanical barrier to further insults from foreign objects as well as to feeding by face flies. Face flies are known to feed on proteinaceous ocular secretions of cattle (Pickens and Miller, 1980). Their sharp prestomal teeth have been shown to cause microscopic damage to the corneal surface (Shugart, 1978). Excessive lacrimation during the initial stages of IBK could attract a larger number of face flies and may delay healing. An eye patch interferes with face fly activity and access to the cornea. On the other hand, eye patches may provide a warm and moist environment that is hospitable to the growth of bacteria including bacteria associated with IBK. A further argument against the use of eye patches is that it is impossible to monitor the progression of a lesion unless the animal is examined in a cattle chute. The manufacturer’s instructions for use indicate that the product will fall off after 7 to 10 d, so the effect may not be comparable under field conditions. In our experience, cattle that were released after receiving an eye patch often lost their patch within minutes after spending time in a holding pen by either rubbing against herd mates or fencing. Attaching the patch with tape helped us to ensure adhesion in most cases. Steers enrolled during the first 2 wk of the trial in the patched group therefore had their patches often replaced at rechecks twice a week.

A systematic review and meta-analysis of antibiotic treatments for IBK found that the best time to observe differences between antimicrobial treatments with corneal ulcer area as the outcome is between days 7 and 10, whereas after 16 d no difference may be seen between antibiotic intervention or placebo (Cullen et al., 2016). Results from our study appear consistent with this finding given the fact that both groups received identical antimicrobial treatments at enrollment and differences between ulcer sizes first became apparent after 14 d following enrollment.

Although we did apply standard criteria for making a diagnosis of IBK at enrollment, it is possible that corneal ulcers that healed within 3 d of initial observation may have been traumatic in origin and misclassified as IBK. However, as assignment status at enrollment was random, any such misclassification would have been unbiased or biased toward the null in regression analysis. In addition, in the field, treatment decisions would be based on initial appearance, not on subsequent healing time. Severity score at diagnosis was a risk factor for healing time, with lower scores resulting in a shorter time to ulcer resolution. Since median time to healing was shorter for all severity scores in the group that received patches vs. the control group, it can be inferred that eye patches may be beneficial even for less severe lesions.

In this study, diagnosis of IBK was based on clinical appearance rather than by recovery of any specific organism from ocular swabs. The swabs taken, however, show that *Moraxella* species were present in this herd. It is not uncommon to see *Moraxella* being isolated from normal appearing eyes, as this genus has been observed as a commensal on ocular surfaces (Lepper and Barton, 1987; Schnee et al., 2015; Bartenslager et al., 2021). The frequency of isolation of *Moraxella* species was higher from lesions, with *Moraxella bovoculi* being the most common species cultured. That *M. bovoculi* was most commonly isolated is consistent with findings of other studies (Loy and Brodersen, 2014; Schnee et al., 2015)

It was surprising that weight gain was higher in steers diagnosed with IBK vs. those that did not develop IBK. Multiple studies have shown that IBK leads to decreases in weight gain, presumably due to the pain associated with IBK that may cause animals to abandon or reduce grazing time until eye lesions start to heal (Killinger et al., 1977; Funk et al., 2014; O’Connor et al., 2019). Our study design involved gathering of steers on a weekly basis, which resulted in disruption of grazing while steers were gathered and moved from pastures to corrals. Steers that were treated for IBK were kept in a paddock close to the cattle examination chute until their lesions had healed; this reduced their travel and pen time during the period they were enrolled in the trial and may have led to the increased weight gain that we observed in pinkeye affected cattle observed.

ADG in patched steers was 0.47 (±0.02) kg vs. 0.43 (±0.02) kg in those without a patch. Although this difference was not statistically significant, those wearing patches had numerically higher gains. Steers that were not wearing a patch spent on average longer in the recovery pen and therefore may have had a nutritional advantage, thus negating potential benefits afforded by an eye patch. However, no statistically significant treatment effect over time (captured in a time by treatment interaction) was found in the mixed model comparing weight gain during the treatment period. Further studies controlling for pasture composition during the entire study period would help us to clarify the effect that pasture could have on weight gain during recovery.

## CONCLUSIONS

For steers wearing an eye patch, corneal ulcers decreased faster during recovery, and healing time from diagnosis to lesion resolution was shorter compared to steers that did not wear a patch. Although we were not able to show a production advantage based on differences in weight gain, weight gain may have been confounded by pasture conditions and the fact that enrolled steers were grazed separately from the rest of the group. The results of this study support the use of a low-cost intervention that may lead to faster recovery from IBK.
